# Accelerated intermittent theta burst stimulation in smoking cessation: No differences between active and placebo stimulation when using advanced placebo coil technology. A double-blind follow-up study

**DOI:** 10.1016/j.ijchp.2022.100351

**Published:** 2022-11-12

**Authors:** Georgios Mikellides, Panayiota Michael, Lilia Psalta, Artemis Stefani, Teresa Schuhmann, Alexander T. Sack

**Affiliations:** a Department of Cognitive Neuroscience, Faculty of Psychology and Neuroscience, Maastricht University, the Netherlands; b Cyprus rTMS Centre, Cyprus; c Medical School, University of Nicosia, Cyprus; d Department of Psychology, University of Cyprus, Cyprus; e School of Science, University of Central Lancashire, Cyprus; f Department of Developmental Neurosciences, Great Ormond Street Institute of Child Health, University College London, United Kingdom; g Department of Psychiatry and Neuropsychology, School for Mental Health and Neuroscience (MHeNs), Brain+Nerve Centre, Maastricht University Medical Centre+ (MUMC+), the Netherlands

**Keywords:** Nicotine dependence, Tobacco craving, Repetitive transcranial magnetic stimulation, Intermittent theta burst stimulation

## Abstract

**Objective:**

This study aims to investigate the longer-term effects of accelerated intermittent theta burst stimulation (aiTBS) in smoking cessation and to examine whether there is a difference in outcome between active and placebo stimulation. The present study constitutes an ancillary study from a main Randomized Controlled Trial (RCT) evaluating the acute effects of aiTBS in smoking reduction.

**Method:**

A double-blind randomized control trial was conducted where 89 participants were randomly allocated to three groups (transcranial magnetic stimulation (TMS)&N group: active aiTBS stimulation combined with neutral videos; TMS&S group: active aiTBS stimulation combined with smoking-related videos; Placebo group: placebo stimulation combined with smoking-related videos). Nicotine dependence, tobacco craving, perceived stress and motivation to quit smoking were measured after completion of 20 aiTBS sessions and during various follow ups (post one week, post one month and post six months).

**Results:**

Our results show that the positive effect on nicotine dependence and tobacco craving that occurred at the end of treatment lasts at least one month post treatment. This effect seems to dissipate six months post treatment. No significant differences were found between the three groups.

**Conclusion:**

Both active and placebo stimulation were equally effective in reducing nicotine dependence and tobacco craving up to one month after the end of treatment.

## Introduction

Tobacco use is a leading cause of various health problems and premature death (World Health Organization, 2021). Nicotine is a highly addictive chemical in tobacco which makes smoking cessation difficult for many smokers (Food and Drug Administration, 2022). In addition to first line tobacco cessation medication such as nicotine replacement therapy (Silagy et al., 2004), alternative and effective treatment methods are offered to support smoking cessation, including noninvasive brain stimulation technologies. Transcranial magnetic stimulation (TMS) is one of these noninvasive brain stimulation techniques representing a versatile intervention that has shown to be clinically effective in various contexts (Klomjai et al., 2015; Mikellides et al., 2021; Pell et al., 2011). Through electromagnetic induction, time-varying magnetic fields are created via an insulated electromagnetic coil placed over a specific area of the scalp (Koutsomitros et al., 2021). These magnetic fields or pulses then pass transcranially through the intact scalp to induce an electric current in the targeted neural tissue (Klomjai et al., 2015; Schilberg et al., 2021). In repetitive transcranial magnetic stimulation (rTMS) electromagnetic pulses are produced repeatedly and can modulate cortical excitability beyond the stimulation period itself (Pell et al., 2011) with low frequency rTMS generally reducing cortical excitability, whereas high frequency rTMS tends to increase cortical excitability of the stimulated brain region (Pell et al., 2011). The more recently introduced Theta Burst Stimulation (TBS) protocols have been shown to be capable of inducing longer lasting neuroplastic changes with intermittent TBS (iTBS) increasing and continuous TBS (cTBS) reducing cortical excitability (Chung et al., 2015; Huang et al., 2005; Schilberg et al., 2017; Thomson et al., 2020). For clinical purposes, TBS is especially interesting, as it provides a much faster brain stimulation intervention, allowing to modulate neuroplasticity based on much shorter stimulation durations as compared to standard rTMS protocols (Schilberg et al., 2017). The accelerate form of iTBS has gained popularity recently, providing multiple sessions within a day to reduce overall treatment duration. An RCT study by Duprat et al. (2016) found that 20 iTBS sessions spread over 4 days at five sessions per day, lead to clinical response in patients with treatment resistant depression (TRD) (Duprat et al., 2016).

The dorsolateral prefrontal cortex (DLPFC) is a frontal brain region involved in executive functions such as inhibitory control as well as reward processing (Feil et al., 2010). These processes are implicated in smoking craving, making DLPFC a potential target region in non-invasive brain stimulation treatments (Yuan et al., 2017). High frequency (HF) - rTMS over the left DLPFC has shown promising results in reducing nicotine craving and cigarette consumption (Amiaz et al., 2009; Hauer et al., 2019; Li et al., 2020). In 2020, the FDA cleared the BrainsWay deep TMS system for its use as an aid in short-term smoking cessation in adults (BrainsWay, 2020). Exposure to smoking related cues has been shown to activate the DLPFC (Brody et al., 2002). Also, the combination of HF- deep TMS treatment and presentation of smoking cues was shown to reduce cigarette consumption with high and long lasting abstinence (Dinur-Klein et al., 2014).

We recently conducted a double-blind randomized control trial to evaluate the effect of active and placebo accelerated intermittent theta burst stimulation (aiTBS) (4 sessions per day for 5 consecutive days) over the left DLPFC in smoking cessation and the impact of smoking-related or neutral cues, during the stimulation, in treatment outcome (Mikellides et al., 2022). The study participants were divided into three groups (TMS&N group: active aiTBS stimulation combined with neutral videos; TMS&S group: active aiTBS stimulation combined with smoking-related videos; Placebo group: placebo stimulation combined with smoking-related videos). Simultaneously with the rTMS treatment, participants were instructed to pay attention to videos that were presented on a monitor placed opposite the treatment chair. Two different forms of videos were used (smoking related videos, e.g. a person smoking cigarette in a restaurant, and neutral videos, e.g. a man cleaning his shoes) in order to either or not induce a state of craving at the time of stimulation. Results showed that the main effect of treatment time was statistically significant, indicating a significant reduction of cigarette consumption, nicotine dependence, craving and perceived stress in all treatment groups, which was maintained for at least a week after the end of treatment. Nevertheless, the type of treatment group and/or the interaction effect between treatment time and treatment group were not statistically significant. Thus, the results showed that both, active as well as placebo stimulation, did not affect immediate treatment outcome (Mikellides et al., 2022).

TMS-induced neuroplastic changes take time to develop and differences between active and placebo stimulation may become stronger and more visible when looking at prolonged effects (Cirillo et al., 2017). The current study investigated, in a double-blind randomized control trial using several follow up measurements, the long-term effects of rTMS in smoking cessation. Also, we examined whether there is a difference in outcome between active and placebo stimulation, combined with smoking-related or neutral cues. We hypothesized that a positive prolonged effect would be observed in the Active TMS groups while relapse would be observed in the Placebo TMS group, because the immediate and acute placebo effects should wear off over time, since no neuroplastic changes should have been induced.

## Methods

### Study design & Participants

Detailed methods of our randomized controlled trial (RCT) have been reported previously (Mikellides et al., 2022). We performed a multi-arm parallel group, double-blinded, randomized, controlled study, where eighty-nine participants were randomly allocated into three groups: the first group received active aiTBS stimulation combined with neutral videos (TMS&N), the second group received active aiTBS stimulation combined with smoking-related videos (TMS&N) and the last group received placebo stimulation (Placebo) combined with smoking-related videos. Participants were aware that there was a 1/3 chance of receiving placebo stimulation but were otherwise blinded to the treatment condition. All patients were TMS naïve and due to the between-subject design had no means to directly compare different TMS conditions.

Eligibility criteria included participants aged 18-70 who were native or fluent Greek speakers. Exclusion criteria included mental objects or implants in the brain, skull or near head (e.g., pacemakers, metal plates), past or current of diagnosis of neurological or psychiatric disorder, use of psychiatric medication, past or current drug or alcohol abuse (other than nicotine), and use of IQOS (“I Quit Original Smoking”) or electronic cigarettes (e-cigarettes).

A total of 89 participants completed the treatment program (60 males; age 45.62 ± 13.42 years). The experiment was carried out at the Cyprus rTMS Center in Larnaca, Cyprus. This study was approved by the Cyprus National Bioethics Committee and written informed consent was obtained from all participants (ΕΕΒΚ/ΕΠ/2019/08) (ClinicalTrials.gov Identifier: NCT05271175).

### Intermittent theta burst stimulation

An accelerated iTBS (aiTBS) treatment comprised 20 sessions in total. Four iTBS sessions were administrated per day, with a 30 minutes break between them, during a 5-day period using a MagPro X100 stimulator (MagVenture, Farum, Denmark). The standardized stimulation localization was over the left DLPFC, determined using the Beam_F3 Locator software (https://www.clinicalresearcher.org/software.htm). A figure-of-eight coil (Coil Cool-B65 A/P) was placed at a 45° angle of the midline over the 10-20 EEG position F3. We applied an iTBS protocol, consisting of triplets of 50Hz that were repeated in a 5Hz rhythm for 2 seconds, followed by an inter train interval of 8 seconds, for a total of 20 trains. A total number of 600 pulses was given per session for 3:08 minutes (Blumberger et al., 2018; Huang et al., 2005). Before the first session, the resting Motor Threshold (rMT) was determined using the Coil C-B60 TMS coil. Stimulation was performed at 100% of rMT.

### Measurements

Participants were asked to complete three self-reported questionnaires:

(1) The Fagerström test for Nicotine Dependence (FTND) (Heatherton et al., 1991) to assess nicotine dependence. The FTND is a short, self-report measure that contains six questions, and the total score is calculated as a sum of these six questions. The total scores of the questionnaire vary from 0 to 10, with lower scores indicating lower dependence on nicotine.
(2) the Tobacco Craving Questionnaire–Short Form (TCQ-SF) (Heishman et al., 2008) to assess tobacco craving. The TCQ-SF is a self-report measure that assesses tobacco craving in four dimensions: emotionality, expectancy, compulsivity, and purposefulness. Each factor scale contains three items. TCQ-SF items were rated on a Likert scale of 1 (strongly disagree) to 7 (strongly agree). Total scores vary from 12 to 84. A high score indicates high tobacco craving.
(3) the Perceived Stress Scale-4 (PSS-4) (Cohen & Williamson, 1988) to assess perceived stress. The PSS-4 is a self-report measure that contains four items which were rated on a Likert scale, ranging from 0 to 4, with those on the positive subscale scored in reverse and the total score being calculated as a sum of these items. The scores vary from 0 to 16, with a higher score indicating higher perceived stress.

Finally, participants were asked to estimate how motivated they were to quit smoking from 0% to 100% using a Visual Analogue Scale.

**Fig. 1:**
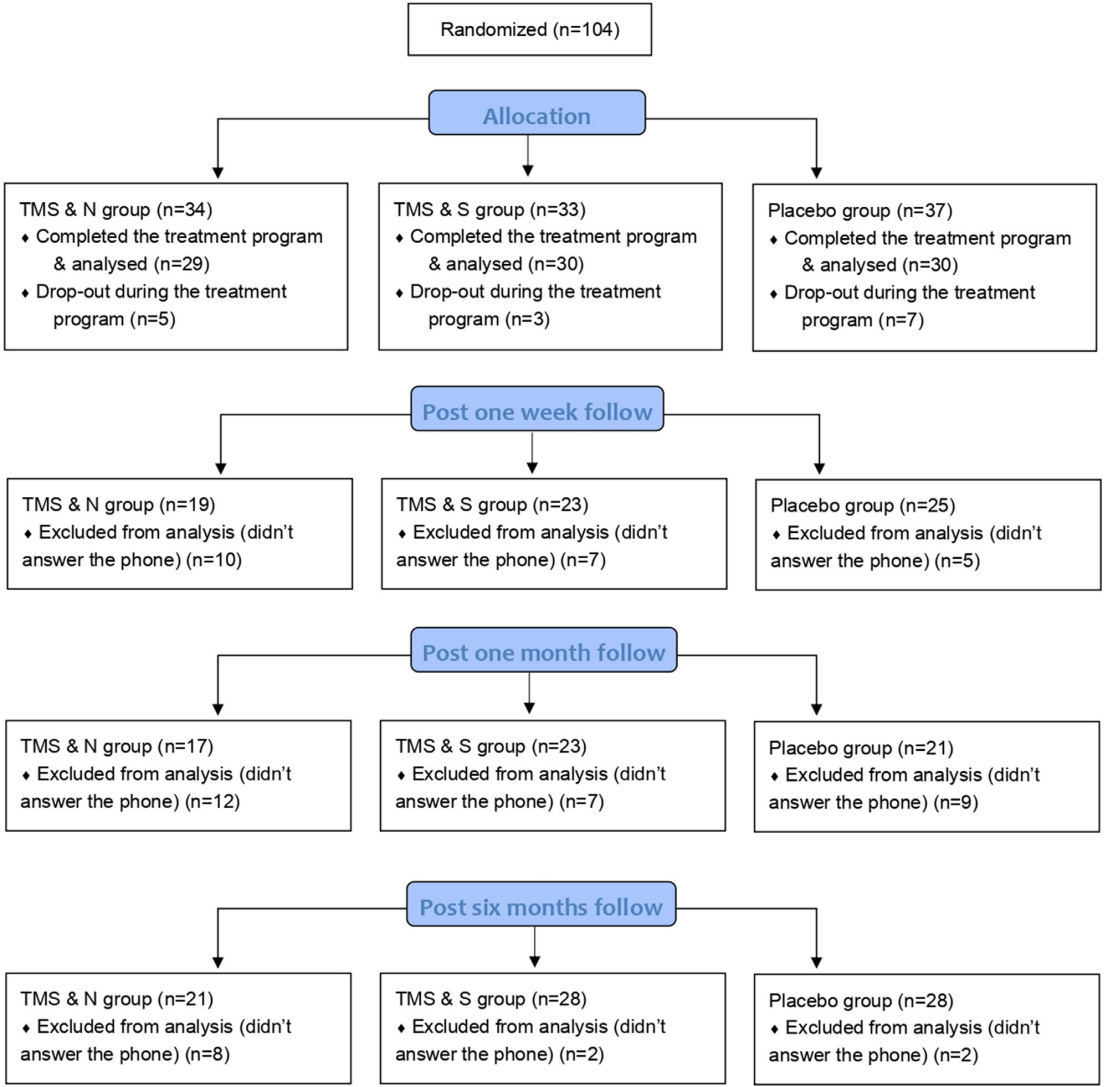
Study flow.

These three questionnaires and the Motivation to quit smoking were administered to participants at baseline, at the end of treatment (on the fifth day), one week post treatment, one month post treatment and six months post treatment. Participants completed the questionnaires by hand at baseline and at the end of the treatment, and then via phone during the follow ups (post one week, post one month and post six months, see Fig. 1). All participants who completed the study (n=89) were asked to complete the post one-week follow up, post one-month follow-up and post six-months follow up, regardless of whether they completed the previous follow ups. No extra sessions of iTBS were performed in any of the follow-up phases.

### Data analysis

SPSS software version 27.0 was used for the statistical analysis of the data (IBM corporation, Endicott, New York). One-way ANOVAs and Pearson chi-square tests were used to test for differences in baseline demographic and smoking-related variables between the completers and dropouts in post one week follow up, post one month follow up and post six months follow up. Mixed factorial ANOVAs were conducted to investigate the effect of both the within factor (Time: end of treatment, post one week follow up, post one month follow up, post six months follow up) and the between factor (Group: TMS&N, TMS&S, Placebo). The dependent variables used for each model were: nicotine dependence, tobacco craving, and perceived stress. Greenhouse–Geisser and Huynh–Feldt degree of freedom corrections were applied to correct for the non-sphericity of the data. Non-parametric tests were used as the variable *Motivation to quit smoking* was not normally distributed at all time-point assessments. Kruskal-Wallis H tests were conducted to compare the mean scores of motivation to quit of the three groups at different timepoints. Non-parametric Friedman Tests were used to determine whether there is a statistically significant difference between the means of the four timepoints. Post hoc Wilcoxon signed-rank tests were conducted to evaluate the significance of mean change in *Motivation to quit smoking* scores between different time points. Missing data were excluded from the final analyses. A significance level was set at α=.05 for all analyses.

## Results

### Study flow

A total of 89 participants completed the 5-day treatment program (60 male and 29 female; age 45.62 ± 13.42 years), of which 59 received active stimulation and 30 received placebo stimulation. Sixty-seven of the participants (65.5% of the TMS & N group, 76.7% of the TMS & S group and 83.3% of the Placebo group) completed the post one-week follow-up, 61 of the participants (58.6% of the TMS & N group, 76.7% of the TMS & S group and 70.0% of the Placebo group) completed the post one-month follow-up and 77 of the participants (72.5% of the TMS&N group, 93.3% of the TMS&S group and 93.3% of the Placebo group) completed the post six-months follow-up. Fig. 1 shows the study flow. Table 1 shows the comparison of the baseline and smoking-related characteristics of dropouts and completers during the follow ups. The data suggests that completers did not differ significantly from dropouts in demographic characteristics across all follow ups (see Table 1). Regarding smoking-related characteristics, completers of post one week follow up were more likely to have quitted smoking in the past compared to dropouts and completers of post six months follow up were more likely to be smokers for more years compared to dropouts. No significant differences were found in the remaining smoking-related variables (Table 1).

**Table 1:** Demographic and smoking related characteristics of completers and dropouts.

	POST ONE WEEK FOLLOW UP			POST ONE MONTH FOLLOW UP			POST SIX MONTHS FOLLOW UP		
Characteristics	Completers	Dropouts	*p* values	Completers	Dropouts	*p* values	Completers	Dropouts	*p* values
	n=67	n=22		n=61	n=28		n=77	n=12	
**Demographic**									
Age (yr)	46.10 ±13.81	44.14±12.31	.554^a^	47.39 ±14.02	41.75±11.29	.065^a^	46.45 ±13.58	40.25±11.37	.137^a^
Gender (M/F)	43/24	17/5	.256^b^	41/20	19/9	.952^b^	52/25	8/4	.953^b^
Education (yr)	13.82±3.73	14.68±3.43	.341^a^	13.85±3.62	14.43±3.77	.493^a^	14.00±3.72	14.25±3.36	.827^a^
Occupation*			.264 b			.683 b			.828 b
Private employee	41 (61.2%)	13 (59.1%)		35 (57.4%)	19 (67.9%)		47 (61.0%)	7 (58.3%)	
Public employee	6 (9.0%)	6 (27.3%)		9 (14.8%)	3 (10.7%)		9 (11.7%)	3 (25.0%)	
Self-employed/ Freelancer	8 (11.9%)	2 (9.1%)		6 (9.8%)	4 (14.3%)		9 (11.7%)	1 (8.3%)	
Unemployed	3 (4.5%)	0 (0.0%)		2 (3.3%)	1 (3.6%)		3 (3.9%)	0 (0.0%)	
Retired	8 (11.9%)	1 (4.5%)		8 (13.1%)	1 (3.6%)		8 (10.4%)	1 (8.3%)	
Student	1 (1.5%)	0 (0.0%)		1 (1.6%)	0 (0.0%)		1 (1.3%)	0 (0.0%)	
**Smoking-related**									
Cigarettes per day	26.73±13.10	32.41±15.29	.094^a^	27.20±13.20	30.18±15.08	.347^a^	28.13±12.83	28.17±19.65	.993^a^
Types of cigarettes*			.353 b			.349 b			.699 b
Normal	50 (74.6%)	15 (68.2%)		47 (77.0%)	18 (64.3%)		57 (74.0%)	8 (66.7%)	
Hand-rolled	15 (22.4%)	5 (22.7%)		12 (19.7%)	8 (28.6%)		16 (20.8%)	4 (33.3%)	
Cigarillos	0 (0.0%)	1 (4.5%)		0 (0.0%)	1 (3.6%)		1 (1.3%)	0 (0.0%)	
Mixed	2 (3.0%)	1 (4.5%)		2 (3.3%)	1 (3.6%)		3 (3.9%)	0 (0.0%)	
Years of smoking	25.62±12.90	23.36±9.72	.454^a^	26.10±13.27	22.82±9.21	.184^a^	25.89±12.66	19.75±6.57	.016^a^
If ever quitted*			.017^b^			.451^b^			.179^b^
No	18 (26.9%)	12 (54.5%)		19 (31.1%)	11 (39.3%)		28 (36.4%)	2 (16.7%)	
Yes	49 (73.1%)	10 (45.5%)		42 (68.9%)	17 (60.7%)		49 (63.6%)	10 (83.3%)	
How many times quitted	1.190±1.28	0.55±0.67	.026^a^	1.05±1.20	1.00±1.19	.858^a^	1.03±1.25	1.08±0.79	.878^a^

Data are means ± standard deviation.

⁎ n(%)

a Independent sample t-test.

b Pearson chi-square test. TMS, Transcranial Magnetic Stimulation.

### Primary outcomes

#### Nicotine dependence

**Fig. 2:**
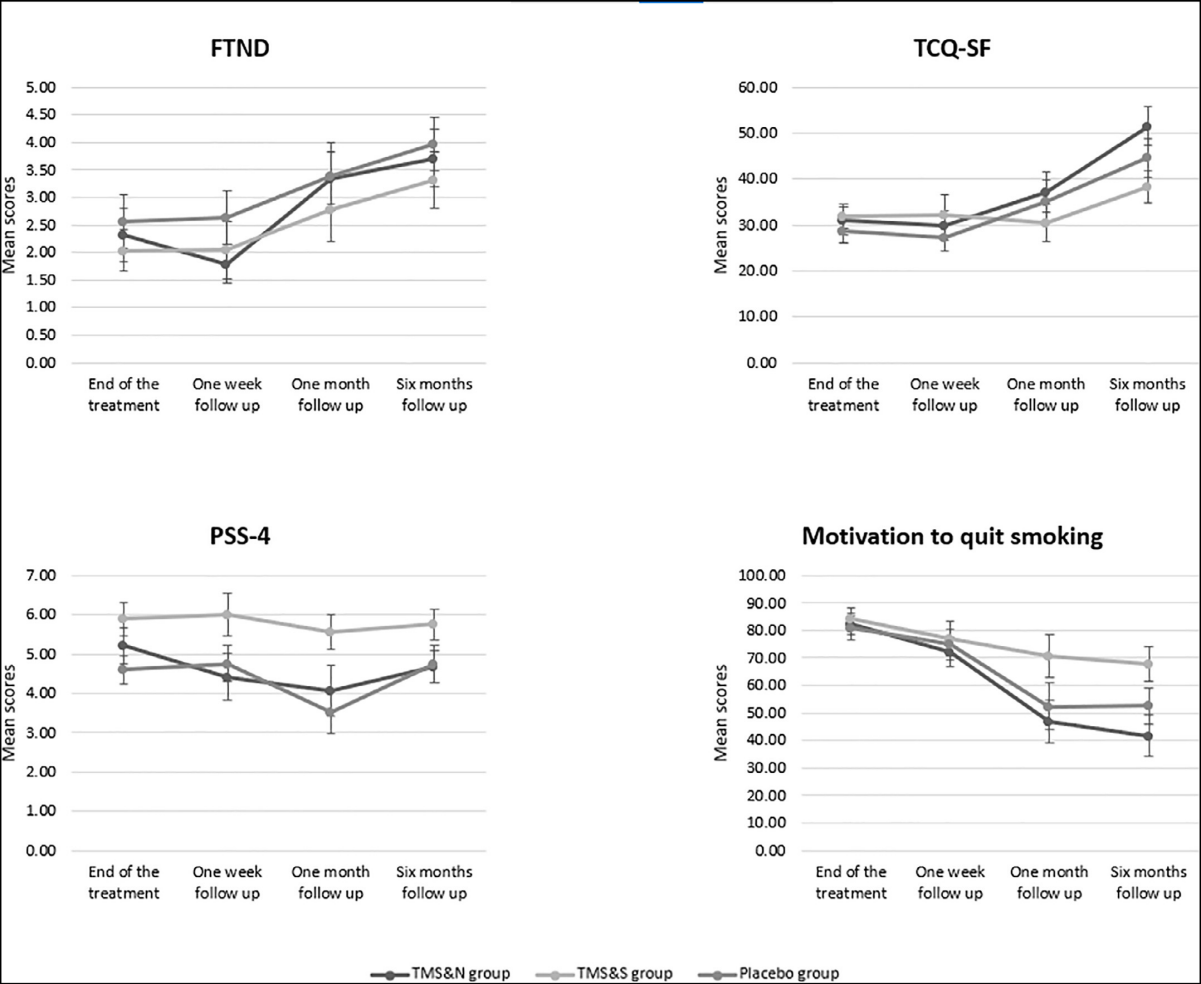
Line graphs showing FTND, PSS-4, TCQ-SF and Motivation to quit smoking scores over time. Data are presented as mean ± SEM.

A 4 (Time: end of treatment, post one week follow up, post one month follow up, post six months follow up) X 3 (Group: TMS&N, TMS&S, Placebo) mixed factorial ANOVA was conducted as measured by the FTND. Mauchly's test indicated that the assumption of sphericity had been violated, *χ^2^(5) =24.693, p<0.001*, therefore degrees of freedom were corrected using Greenhouse-Geisser of sphericity (ε=.774). The interaction effect between Time and Group was not statistically significant, *F(4.642,97.473)=.478, p=.778, η_p_^2^=.022.* There was a statistically significant main effect of Time, *F(2.321,97.473)=11.153, p<0.0001, η_p_^2^=.21*0 but no significant effect of Group, *F(2,42)=.673, p=.516, η_p_^2^=.031* (see Table 2 for means and standard deviations) (Fig. 2). Post-hoc pairwise comparisons using the Bonferroni correction revealed that nicotine dependence was significantly increased in post one month follow up (M=2.87, SD=2.67) compared to post one week follow up (M=2.02, SD=2.32) and in post six months follow up (M=3.58, SD=2.78) compared to the end of treatment and post one week follow up (M=2.02, SD=2.32) (Table 3).

**Table 2:** Means and standard deviations.

	End of treatment	Post one week follow up	Post one month follow up	Post six months follow up
**FTND**				
TMS & N	2.31 (2.62)	1.79 (1.55)	3.35 (1.97)	3.71 (2.43)
TMS & S	2.03 (2.08)	2.04 (2.50)	2.78 (2.80)	3.32 (2.74)
Placebo	2.57 (2.69)	2.64 (2.41)	3.38 (2.84)	3.96 (2.57)
**TCQ-SF**				
TMS & N	30.93 (17.05)	29.84 (14.18)	37.06 (17.94)	51.52 (19.50)
TMS & S	31.97 (14.61)	32.17 (21.57)	30.61 (19.63)	38.32 (18.22)
Placebo	28.63 (13.10)	27.28 (14.82)	35.24 (20.63)	44.68 (22.34)
**Motivation to quit smoking**				
TMS & N	82.41 (20.59)	72.37 (23.41)	47.06 (31.72)	41.67 (34.76)
TMS & S	84.26 (19.79)	77.17 (30.07)	70.65 (37.43)	67.86 (32.53)
Placebo	80.83 (23.38)	75.00 (27.95)	52.38 (38.65)	52.68 (34.92)

Data are means (standard deviation), averaged over all participants per group. FTND, Fagerström test for Nicotine Dependence; TCQ-SF, Tobacco Craving Questionnaire–Short Form.

**Table 3:** Pairwise comparisons.

	FTND			TCQ-SF		
Pairs	Mean change	Standard Error	*p* value	Mean change	Standard Error	*p* value
EndOfTreatment -PostOneWeekFollowUp	-.001	.270	1.000	1.466	2.174	1.000
EndOfTreatment -PostOneMonthFollowUp	.882	.361	.113	4.919	2.855	.553
EndOfTreatment -PostSixMonthsFollowUp	1.554	.391	.002	16.402	3.588	<0.0001
PostOneWeekFollowUp -PostOneMonthFollowUp	.882	.222	.002	3.453	2.069	.616
PostOneWeekFollowUp -PostSixMonthsFollowUp	1.555	.343	<0.0001	14.936	3.629	.001
PostOneMonthFollowUp -PostSixMonthsFollowUp	.673	.300	.183	11.483	3.345	.008

FTND, Fagerström test for Nicotine Dependence; TCQ-SF, Tobacco Craving Questionnaire–Short Form. p<0.05. Adjustment for multiple comparisons: Bonferroni.

#### Tobacco craving

A 4 (Time: end of treatment, post one week follow up, post one month follow up, post six months follow up) X 3 (Group: TMS&N, TMS&S, Placebo) mixed factorial ANOVA was conducted as measured by the TCQ-SF. Mauchly's test indicated that the assumption of sphericity had been violated in both situations, *χ^2^(5) =25.824, p<0.001*, therefore degrees of freedom were corrected using Huynh–Feldt of sphericity (ε=.821). The interaction effect between Time and Group was not statistically significant, *F(4.925,103.435)=1.042, p=.397, η_p_^2^=.047*. There was a statistically significant main effect of Time, *F(2.463,103.435)=12.175, p<0.0001, η_p_^2^=.225.* However, there was no significant effect of Group, *F(2,42)=.643, p=.531, η_p_^2^=.030* (see Table 2 for means and standard deviations) (Fig. 2). Post-hoc pairwise comparisons using the Bonferroni correction indicated that tobacco craving was significantly increased in the post six months follow up (M=44.11, SD=22.64) compared to the end of the treatment (M=28.33, SD=15.57), post one week follow up (M=29.13, SD=18.45) and post one month follow up (M=32.60, SD=19.62) (Table 3).

### Secondary outcomes

#### Perceived stress

A 4 (Time: end of treatment, post one week follow up, post one month follow up, post six months follow up) X 3 (Group: TMS&N, TMS&S, Placebo) mixed factorial ANOVA was conducted as measured by PSS-4. The interaction effect between Time and Group, *F(6,126),=.510, p=.800, η_p_^2^=.024,* as well as the main effect of Time, *F(3,126)=1.187, p=.317, η_p_^2^=.027,* and the effect of Group, *F(2,42)=1.389, p=.260, η_p_^2^=.062,* were not statistically significant (Fig. 2).

#### Motivation to quit smoking

Kruskal-Wallis H tests showed that there were no statistically significant differences in Motivation scores between the three groups during all the time points, except of the post six months follow up where there was a statistically significant difference, *H(2)=6.803, p=.033*, with a mean rank of 30.74 for TMS&N group, 46.88 for TMS&S group and 37.32 for Placebo group. Comparison of the repeated measures was performed using Friedman's test showing a statistically significant difference across the sample, *χ^2^(3)=18.079, p= p<0.0001* (Fig. 2)*.* Post-hoc analysis with Wilcoxon signed-rank tests were conducted, to evaluate the significance of mean change in Motivation to quit smoking scores between different time points. A significant decrease was seen in all the pairs, except of the pair post one month follow up vs post six months follow up where no statistically significant differences were found (Table 4).

**Table 4:** Results of Wilcoxon signed-rank tests for Motivation to quit smoking scores.

Pairs	Mean change	Z value	*p* value
EndOfTreatment - PostOneWeekFollowUp	-8.33	-3.136	.002
EndOfTreatment - PostOneMonthFollowUp	-25.86	-4.461	<.0001
EndOfTreatment - PostSixMonthsFollowUp	-28.38	-5.058	<.0001
PostOneWeekFollowUp - PostOneMonthFollowUp	-13.02	-2.633	.008
PostOneWeekFollowUp - PostSixMonthsFollowUp	-18.10	-3.149	.002
PostOneMonthFollowUp - PostSixMonthsFollowUp	-2.59	-.460	.646

## Discussion

This study sought to evaluate the long-term effects of aiTBS in smoking cessation. We hypothesized that active TMS leads to a positive prolonged effect compared to placebo TMS. However, the findings of the current study do not support this hypothesis. In fact, the positive effect of the treatment on nicotine dependence and tobacco craving lasted at least up one month post treatment, independent of the treatment condition. After 6 months, this effect was gone in all groups.

The findings of the current study do not support previous findings. Earlier studies using HF-rTMS over the left DLPFC, have found a significant reduction of cigarette consumption in active TMS groups compared to placebo TMS groups at the end of treatment, which lasted three weeks up to one month post treatment (Huang et al., 2016; Li et al., 2020; Prikryl et al., 2014). Also, the outcome of the present study is contrary to that of recent study who found a reduction in nicotine dependence and tobacco craving at the end of treatment in both the HF-rTMS active and placebo groups, but during the 3 months follow up this improvement was persistent only in the active group (Abdelrahman et al., 2021). On the other hand, our post 6 months follow up results are in line with those of Amiaz et al. (2009), where no significant differences were found between active and placebo TMS groups in nicotine dependence and craving six months post treatment. Finally, it is clear from the results that the type of video did not affect the treatment outcome. This result is consistent with a previous study showing that exposure to smoking-related cues had no effect on nicotine consumption and nicotine dependence (Amiaz et al., 2009).

Contrary to the expectations, all treatment conditions lead to significant reduction in nicotine dependence and tobacco craving, which lasted at least one month post treatment. This pattern of results is consistent with previous literature on chronic headaches and post-stroke rehabilitation, which reports that the placebo effect of rTMS treatment persists at least 3 months after treatment (Granato et al., 2019; Jin et al., 2021). Large placebo effects appear in pharmacological as well as in neurostimulation and surgical trials (Brunoni et al., 2009; Vase & Wartolowska, 2019). Placebo effects are a very common phenomenon in TMS practice and can be considerably large and even equal to the effect achieved with active TMS stimulation, which may influence the clinical results obtained with TMS (Kaptchuk et al., 2000; Mansur et al., 2011; Razza et al., 2018). Several factors of a TMS treatment may contribute to enhanced placebo effects such as the interaction with the TMS technician, the TMS device, or realistic placebo coils (Burke et al., 2019).

Different placebo TMS approaches can be used to achieve a placebo condition in TMS, aiming to mimic the auditory and somatosensory experience of active TMS without brain stimulation (Duecker & Sack, 2015). The use of electrical stimulation in combination with a placebo TMS coil has been reported as an effective TMS approach to achieve placebo condition (Duecker & Sack, 2015). In Mikellides and colleagues study (2022), both, active and placebo, stimulation were performed using an advanced double blind placebo stimulation technology, the figure-of-eight coil (Coil Cool-B65 A/P), which is capable to mimic both, the visual and auditory experience of active TMS as well as similar somatosensory skin sensation. Using a low intensity current stimulator built into the coil and a pair of surface electrodes placed just below the hairline on the scalp of each participant, this coil is designed to support true “double blinded” clinical trials as it can produce active and placebo stimulation by flipping it. The use of a novel and advanced placebo coil technology in Mikellides et al (2022) study may contribute to the fact both active and placebo stimulation were highly effective in reducing cigarette consumption and craving.

The placebo effect was also shown to be larger in more intense TMS protocols and especially accelerated protocols (Baeken et al., 2013). The current study confirmed the findings of Baeken (2018), reporting no clinical differences between the placebo and active accelerated treatments (accelerate HF-rTMS and accelerate iTBS) in refractory MDD patients (Baeken, 2018). In a similar vein, no statistically significant effects were found between placebo and active stimulation on depression severity symptoms following aiTBS (Duprat et al., 2016). On the contrary, the Stanford neuromodulation therapy (SNT) protocol, an FDA cleared, accelerated iTBS protocol, was found to be more effective compared to placebo stimulation in treatment resistant depression (TRD) (Cole et al., 2022). It is currently unclear why the SNT protocol showed so small placebo effects as compared to other studies. Maybe the small and selective sample or the very short treatment duration of 5 days only in the SNT study contributed to this discrepancy.

In the present study, nicotine dependence and tobacco craving increased at post 6 months follow up. The observed increase could be attributed to the outbreak of the COVID-19 pandemic that started in March 2020 and included prolonged periods of lockdown mandates. Almost all data of post 6 months follow up were obtained in the period April-June 2020, a period related to the first lockdown in Cyprus, which was followed by strict guidelines and measures (Stylianou et al., 2020). As mentioned in the literature, the COVID-19 lockdown was associated with an increase in cigarette consumption in European countries as a result of the social isolation (Malta et al., 2021; Vanderbruggen et al., 2020). Nevertheless, the outbreak of COVID-19 may be a possible factor for the increase in nicotine dependence and tobacco craving in the present study, but this cannot be statistically substantiated. Another possible explanation for this increase during the post 6 months follow up may be the absence of maintenance sessions after the completion of the treatment period. Maintenance after a successful response to rTMS treatment can contribute in preventing relapse (Rachid, 2018). However, the optimal stimulus parameters for maintenance rTMS remain unclear (Rachid, 2018).

Some potential limitations of this double-blind follow-up study need to be acknowledged. For instance, participants were not asked to avoid receiving any other form of smoking cessation treatments or interventions during the follow-ups. Therefore, we have no information on whether their clinical condition remained stable throughout the follow-up period and whether our findings are due to the rTMS treatment alone. Secondly, cigarette consumption in numeric values was not measured during the follow up phase. Additionally, all data were collected via phone calls through self-reported questionnaires which may have affected the accuracy and reliability of the assessment. We did not use objective measures of nicotine consumption (e.g., breath carbon monoxide meter device) during the follow ups.

## Conclusion

In summary, our results demonstrate that both active and placebo stimulation were equally effective in reducing nicotine dependence and tobacco craving up to one month after the end of treatment. Placebo effect should be considered a major source of bias in the assessment of rTMS efficacy.

## Declaration of Interest

None.
